# The Panoramic Landscape of Human Suffering

**DOI:** 10.3201/eid1311.000000

**Published:** 2007-11

**Authors:** Polyxeni Potter

**Affiliations:** * Centers for Disease Control and Prevention, Atlanta, Georgia, USA

**Keywords:** Human suffering, Pieter Bruegel the Elder, landscape painting, emerging infections, art and humanities, about the cover

**Figure:**
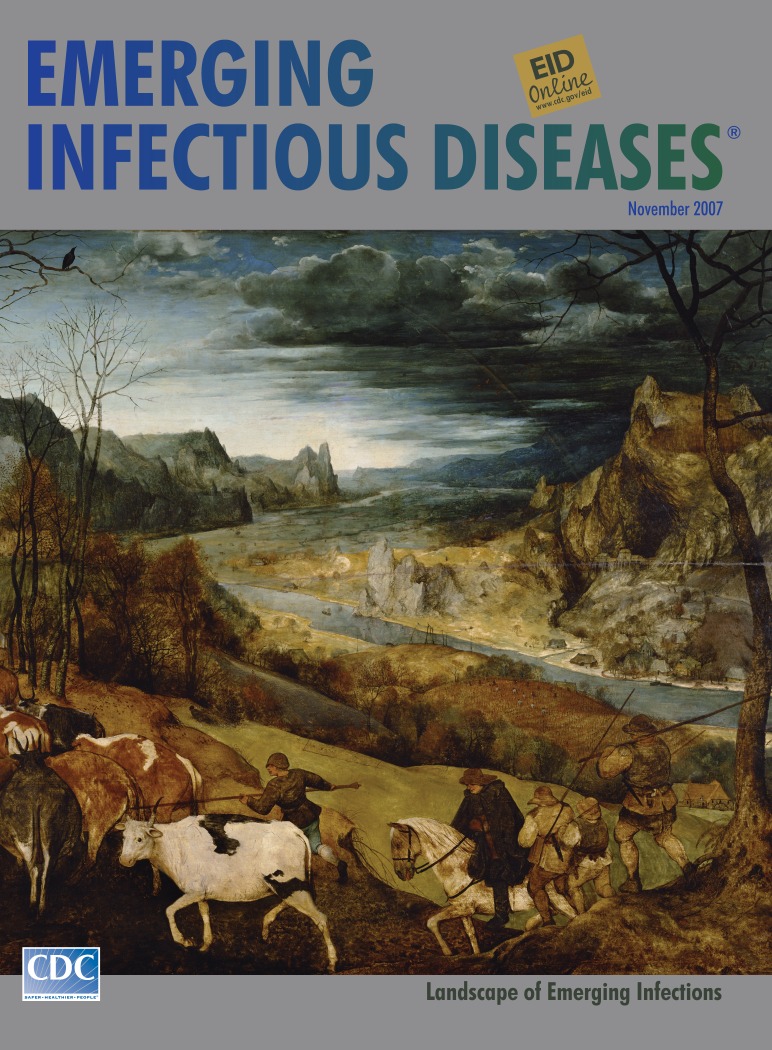
**Pieter Bruegel the Elder (c. 1525–1569) From The Seasons (1565), Return of the Herd.** Oil on panel (160 cm × 120 cm). Kunsthistorisches Museum, Vienna, Austria

The Old Masters were never wrong about suffering, wrote W.H. Auden. They understood how it takes place, “While someone else is eating or opening a window or just walking dully along” (*1*). Auden was referring to the work of Pieter Bruegel the Elder, which dwelled on suffering, along with labor and merrymaking, the lot of simple folk. He painted them with such dedication it earned him the title “Peasant Bruegel.”

He so delighted in the behavior of peasants, he disguised himself as one, and went out into the countryside to mingle with them during their feasts and weddings, “... brought gifts like the other guests, claiming relationship or kinship with the bride or groom.” He observed “how they ate, drank, danced, capered, or made love, all of which he was well able to reproduce cleverly and pleasantly,” wrote chronicler Karel van Mander, “... men and women of the Campine and elsewhere―naturally, as they really were” (*2*). So well did he represent them and through them all of humanity, that in the words of his friend the famed cartographer Abraham Ortelius, “he painted many things that cannot be painted” (*3*).

He was held in high esteem by scholars of his day, among them poet and engraver Dierick Volckherzoon Coornhert, who once was so impressed by Bruegel’s work, he wrote, “I examined it with pleasure and admiration from top to bottom for the artistry of its drawing and the care of the engraving…methinks I heard moaning, groaning and screaming and the splashing of tears in this portrayal of sorrow” (*3*).

What we know about the artist comes from Karel van Mander’s Painter’s Book, published in 1604, some 35 years after Bruegel’s death. He was likely born in the late 1520s in Breda (modern Netherlands); lived and worked in Antwerp and Brussels; and apprenticed with sculptor, architect, painter, designer of tapestry and stained glass Pieter Coecke van Aelst, whose daughter he later married. The apprenticeship had little influence on his style but did introduce him to humanist circles and the work of Maria Verhulst Bessemers, his mother-in-law, a skilled miniaturist and illuminator who experimented with tempera on linen (*4*).

After 1559, he dropped the “h” from his name, though his sons, Jan and Pieter the Younger, retained the original Brueghel spelling. Too young at the time of his death to learn from their father, the sons studied with their grandmother and became important artists in their own right, part of a brilliant legacy of four generations in the 16th and 17th centuries.

Like many northern artists, Bruegel traveled to Italy. He visited Naples and Messina and lived in Rome, where he worked with Giulio Clovio, the “prince of miniaturists” according to Giorgio Vasari. Inspired by Italian landscape painting, he “did many views from nature so it was said of him when he traveled through the Alps that he had swallowed all the mountains and rocks and spat them out again, after his return, onto his canvases and panels, so closely was he able to follow nature there and in his other works” (*3*). Drawings of the Alpine landscape published as engravings when he returned to Antwerp brought him early fame. They were completed during his long association as draftsman for leading print publisher Hieronymus Cock.

Along with drawing and designing for Cock’s engravings, Bruegel continued to paint. He favored multifigure compositions in which groups were seen from above. Some of his paintings recalled the fantastic landscapes of the ever popular Hieronymus Bosch (c. 1450–1516). So successful was the resemblance that humanist Domenicus Lampsonius complimented Bruegel by calling him “a second Bosch.” But the Master’s interest in the burlesque was brief.

Bruegel came to landscape painting from the tradition of Joachim Patinir and the Netherlandish painters, inventors of the genre, and from the Venetians, whose work so impressed him. But his genius went far beyond these. His compositions, carefully structured and realistic, were spare, ahead of their time in their focus on shape and movement. Intrigued by the workings of nature, he turned away from idealized landscapes. Familiar with the common people, he translated moralizing and proverbial tales into vernacular earthy scenes infused with humor and whimsy. “There was always more than he painted” (*3*).

Landscape painting has been linked to the rise of Antwerp. The city on the Schelde was a prosperous commercial and publishing center. Demand for luxury goods created a flourishing art market, for as Karel van Mandel put it, “art gladly resides with wealth” (*5*). Antwerp’s guild, in which Bruegel was accepted as Master, boasted 300 artists, at a time when the city supported 169 bakers, 78 butchers, and 75 fishmongers. Landscapes were painted for the open market, and prints were big business.

Unlike many of his contemporaries who struggled to compete, Bruegel was patronized by connoisseurs and earned fame and prestige during his lifetime. Wealthy merchant Niclaes Jongelinck owned 16 of his works. On commission, Bruegel painted for Jongelinck’s home a series representing the seasons. Five of likely six panels survive, among them, Return of the Herd, on this month’s cover. Though created in the medieval tradition of calendar scenes, each panel focused not on the labors of the season alone but on the transformations of nature and its interrelationships with humans.

The Seasons represents the mature work of a man called by his contemporaries “the most perfect artist of the century” and contains many innovations used to express weather conditions, light effects, and human behavior (*3*). Symbolic color was used to invoke seasonal atmosphere. Precise execution gave way to faster, sketchier, more spontaneous technique, allowing greater naturalness and expression in the figures. Paint was thinner to let underpaint show. Peter Paul Rubens later studied this technique.

Return of the Herd has a circular rhythm linking the foreground with the background, the ritual return of the herd with mountains and gathering clouds. The high-horizon vista of trees, running water, and hills dominates. Yet, tempered by the presence of unvarnished humanity, the cosmographic vision turns parochial. Winter is just around the corner. Humans and animals head for cover.

“Nature herself feared being outdone by Bruegel” (*3*). But his fertile metaphorical terrain, with its rich tonal variations, rhythmical movement, and insuppressible aura of death and regeneration, without parallel in the 16th century, may have found its match in our times. The landscape of emerging infections in our November issue, broad, diverse, and fueled by human activity, rivals Bruegel’s in geographic expanse, topographical detail, and the threat of unrelenting human hazard: antimicrobial drug resistance, violent conflict, HIV/AIDS, vectorborne infections, epizootic (H5N1) and pandemic flu, ecologic disasters, community- and hospital-acquired infections. Human suffering, as Auden put it, so well understood by the Old Masters, continues unabated, “... in a corner, / some untidy spot / where the dogs go on with their doggy life and the torturer’s horse / scratches its innocent behind on a tree” (*1*).
